# Single-Gelator Structuring of Hemp Oil Using Agarose: Comparative Assembly, Electronic Nose Profiling, and Functional Performance of Hydroleogels Versus Oleogels in Shortbread Cookies

**DOI:** 10.3390/polym17141988

**Published:** 2025-07-20

**Authors:** Oliwia Paroń, Joanna Harasym

**Affiliations:** 1 Department of Biotechnology and Food Analysis, Wrocław University of Economics and Business, Komandorska 118/120, 53-345 Wrocław, Poland; oliwia.paron@ue.wroc.pl; 2 Adaptive Food Systems Accelerator–Science Centre, Wrocław University of Economics and Business, Komandorska 118/120, 53-345 Wrocław, Poland

**Keywords:** agarose, oleogel, hydroleogel, hemp oil, fat replacement, shortbread, electronic nose, texture, water activity

## Abstract

This study demonstrates an innovative single-gelator approach using agarose (1% and 2% *w/w*) to structure cold-pressed hemp oil into functional fat replacers for shortbread cookies, achieving a 40% reduction in saturated fatty acids compared to butter. Comprehensive characterization revealed that hydroleogels exhibited superior crispiness (45.67 ± 3.86 N for 2% agarose hydroleogel—HOG 2%) but problematic water activity (0.39–0.61), approaching microbial growth thresholds. Conversely, oleogels showed lower crispiness (2.27–3.43 N) but optimal moisture control (aw = 0.12–0.16) and superior color stability during 10-day storage. Electronic nose analysis using 10 metal oxide sensors revealed that oleogel systems preserved characteristic aroma profiles significantly better than hydroleogels, with 2% agarose oleogel (OG 2%) showing 34% less aroma decay than pure hemp oil. The 2% agarose oleogel demonstrated optimal performance with minimal baking loss (5.87 ± 0.20%), excellent structural integrity, and stable volatile compound retention over storage. Morphological analysis showed that hemp oil cookies achieved the highest specific volume (2.22 ± 0.07 cm^3^/g), while structured systems ranged from 1.12 to 1.31 cm^3^/g. This work establishes agarose as a versatile single gelator for hemp oil structuring and validates electronic nose technology for the objective quality assessment of fat-replaced bakery products, advancing healthier food design through molecular approaches.

## 1. Introduction

The growing demand for healthier baked goods has accelerated research into saturated fat reduction strategies, particularly through lipid structuring technologies. Emulsion-filled gels (EFGs)—biphasic systems featuring oil droplets immobilized in gelled aqueous phases—have emerged as effective fat mimetics in shortbread cookies. Studies demonstrate that inulin-based EFGs with extra virgin olive oil (EVOO) can replace up to 50% of butter while enabling “reduced saturated fat” claims (≥30% reduction) under EU regulations (Regulation EC No 1924/2006) [1,2]. For instance, 40–50% EFG substitution reduces saturated fat by 37.5–54.2 g/100 g, significantly improving nutritional profiles without compromising oxidative stability during storage [3].

Oleogels represent another promising approach, structuring liquid oils (e.g., hemp seed oil) into solid-like networks using gelators like waxes or monoglycerides. These systems mimic the rheological properties of traditional fats while enhancing polyunsaturated fatty acid (PUFA) content [4,5]. Recent innovations combine oleogels with hydrogels to form bigels, which offer synergistic advantages: improved mechanical stability, controlled water/oil interactions, and potential for bioactive compound delivery [6]. Biphasic gelation (bigel) technology has emerged as a novel strategy for structuring liquid oils into solid-like fats without relying on high SFA. Bigels combine oleogelation (OG) and hydrogelation (HG) to entrap both oil and water phases within a three-dimensional network [7]. These systems exhibit solid-like mechanical properties comparable to commercial fats but feature improved SFA:UFA profiles due to the liquid oil component [8,9]. Critically, bigels synergize the advantages of their individual phases: the OG imparts mechanical strength while the HG enhances resistance to permanent deformation [10,11,12]. Recent work by Nutter et al. [12] developed plant-based bigels using rice bran wax (RBW)/soybean oil OG and an alginate/κ-carrageenan HG. These bigels demonstrated rheological properties akin to bakery fats, offering a clean-label (emulsifier-free), plant-based alternative high in PUFA.

Despite these advances, the performance of plant-based oleogels, especially those leveraging nutrient-dense oils like cold-pressed hemp oil, remains underexplored in baked goods. Hemp oil is rich in ω-3/6 PUFAs and antioxidants, making it ideal for developing functional cookies with enhanced lipid quality and potential health benefits [13].

A critical challenge in fat replacement is preserving sensory attributes. EFG studies reveal that exceeding 50% butter substitution alters texture and volatile profiles: higher EFG levels increase hardness (due to gluten development from elevated water availability) and darken color (via inulin-mediated Maillard reactions) [1]. Total butter replacement (100%) with EFGs drastically reduces fracture stress and buttery flavor compounds (e.g., methyl ketones), lowering consumer acceptance [3]. Similarly, volatile profiles shift toward lipid oxidation products (e.g., hexanal) when butter is fully replaced [3]. These results confirm the need to balance nutritional improvements with microstructure and flavor preservation, a gap this study addresses using hemp-based gels. Despite advances in bigel design, few studies have evaluated their performance as solid fat replacers in food matrices [9,14], with minimal research on shortbread products [8]. Existing studies often focus on model systems or partial fat replacement, leaving gaps in understanding how bigels behave in high-fat (≥30% *w/w*), low-moisture applications like shortbread.

Hemp seed oil emerges as an ideal candidate for structured lipid systems due to its balanced ω-6/ω-3 polyunsaturated fatty acid (PUFA) ratio and high antioxidant content. When incorporated into oleogels or bigels, it not only reduces saturated fats but also introduces potential health benefits, including anti-inflammatory and cardiovascular protective effects [13]. However, successful integration into baked goods requires addressing challenges in product quality. Studies reveal that high replacement levels (>50%) of butter with bigels in shortbread cookies increase hardness (up to 43.33 N) and baking loss (up to 19.34%) while reducing spreadability and lightness (*ΔE* > 3) due to moisture retention and restricted fat melting [15]. These changes correlate with heightened gluten network formation and limited dough flow during baking, a trade-off that necessitates careful formulation balancing.

This study aims to develop and characterize shortbread cookies in which traditional fats are replaced with hemp-seed-oil-based oleogels and hydroleogels (hydroleogels are hybrid emulgels consisting only of water, oil, and polymer oleogelators). The research focuses on the physicochemical properties, texture, color, water activity, and volatile compound profiles assessment of the cookies immediately after baking and after ten days of storage, specifically evaluating agarose-structured hemp oil systems (both HOG and OG) as full (100%) butter replacers in shortbread.

The replacement of traditional dairy fats in baked goods represents a significant challenge in food technology, particularly regarding the preservation of characteristic volatile compounds that define product quality and consumer acceptance. This study employed PEN3 electronic nose technology to comprehensively analyze volatile emission patterns from shortbread cookies formulated with butter, cold-pressed hemp oil, and novel gel-based fat replacement systems. The experiment assessed six distinct formulations that differ in fat type: traditional butter as the control, pure hemp oil, hydroleogels containing 1% and 2% agarose (HOG 1%, HOG 2%), and corresponding oleogels (OG 1%, OG 2%) prepared through hydroleogel freeze-drying and shearing.

## 2. Results and Discussion

### 2.1. Textural Properties of Cookie Doughs

Texture plays a key role in the processing characteristics of cookie doughs, especially shortbread. The consistency impacts not only handling but also the structural and sensory quality of the final product. The difference in the texture of shortbread doughs evaluated using a double compression test at two time intervals, 5 and 15 min after removal from refrigeration, revealed the room temperature handling properties (Table 1).

Butter-based dough exhibited the highest initial hardness (37.30 ± 1.92 N at 5 min), significantly higher than all other fat systems (*p* < 0.05), due to the solid state of butter at low temperatures. Hemp oil dough showed the lowest hardness (3.87 ± 0.95 N at 5 min), resulting in a soft, highly pliable matrix. Among structured fat systems, OG 2% and HOG 2% achieved intermediate hardness values (9.08 ± 0.48 N and 8.35 ± 1.61 N, respectively), indicating better structuring due to increased agarose content. Over time, hardness declined across all samples, most notably in the butter-based dough (from 37.30 N to 16.33 N), indicating significant thermal softening. OG 2% retained more of its structure (from 9.08 N to 7.72 N), showing the best stability. This suggests that oleogels with higher structuring agent content better resist thermal softening, which is a trend consistent with previous studies [16].

Cohesiveness was highest in hydroleogel systems (e.g., HOG 1% at 0.34 ± 0.12), while butter and all oleogel-based doughs remained significantly lower (0.06–0.10), indicating weaker internal bonding. This could be due to water–polymer interactions in the hydrogel phase, enhancing network cohesion [17]. Springiness was also greater in hydroleogels containing doughs (HOG 1% and HOG 2% = 0.32–0.37), while oil-only and butter systems showed much lower values (0.09–0.26). These properties may contribute to a more elastic handling behavior in HOG systems, which could impact shaping and sheeting during processing.

Butter-based doughs showed the highest gumminess (3.47 ± 0.55 N at 5 min), followed by HOG 2% (1.86 ± 0.27 N). Doughs made with hemp oil showed minimal gumminess (0.14–0.21 N), which reflects their weak structure. Oleogel-based doughs (OG 1%, OG 2%) ranged between 0.55 and 0.65 N and showed a good balance of cohesiveness and resistance, desirable for forming. Resilience followed a similar trend: butter showed the highest initial values (1.86), while oleogels showed improved recovery capacity over time, particularly OG 2% (from 1.07 to 1.67), indicating improved structure retention and viscoelastic recovery.

Notably, doughs with 2% agarose-based oleogels (OG 2%) demonstrated improved firmness compared to their 1% counterparts, suggesting that higher concentrations of agarose enhance the structuring ability of oleogels. This observation aligns with previous findings where increased gelator concentrations in oleogels led to firmer doughs, closely resembling the textural properties of traditional fat-based doughs [4].

Overall, the structuring potential of agarose-based oleogels, especially at 2% concentration, proved most effective in replicating the mechanical functionality of butter. These systems not only provided moderate hardness and good resilience but also maintained textural stability over time. Hydroleogels exhibited superior cohesiveness and elasticity but were more sensitive to temperature-induced changes. These results support the feasibility of using structured hemp oil systems as functional fat replacers in cookie doughs.

### 2.2. Color Analysis of Fats, Doughs, and Cookies

Color is a key sensory attribute that influences consumer perception and product acceptability. This study evaluated the color properties of three material stages: raw fats, raw cookie doughs, and baked cookies (immediately after baking and after 10 days of storage).

As shown in Table 2, raw fats exhibited substantial differences in color. Butter showed the highest lightness (L* = 92.58 ± 0.17), consistent with its pale-yellow appearance, while cold-pressed hemp oil (L* = 44.79 ± 0.97) and its structured forms (HOGs and OGs) were significantly darker (*p* < 0.05). Color saturation (C*) and hue values (h°) also varied widely: hydroleogels (HOG 1% and HOG 2%) had high b* values (26.9, 27.4), indicative of strong yellowness.

When incorporated into dough, these pigments are partially transferred. Doughs made with OG 1% and OG 2% retained lower L* values (~53–56) and higher b* (23–25), while butter-based dough remained brighter (L* = 80.45 ± 2.04) and more neutral in tone.

The a* and b* patterns suggest stronger pigment diffusion from oleogels than from hydroleogels, likely due to the weaker structural entrapment of colored compounds in the oleogel matrix [4].

**Figure 1:**
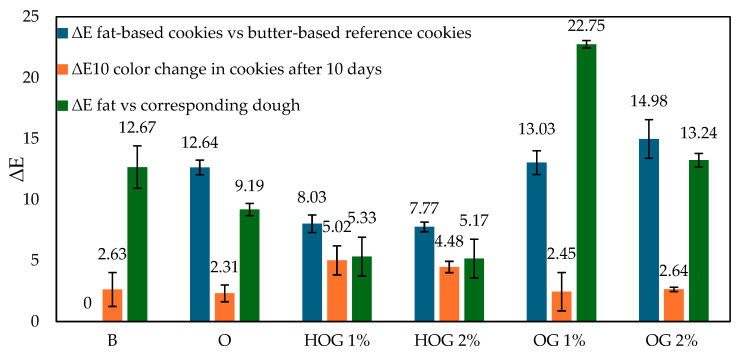
Color differences (∆*E*) in fat-based cookies and dough samples.

The ∆*E* values between fats and their corresponding doughs (Figure 1, green bars) confirmed this observation. The smallest shift was observed in HOG 1% (∆*E* = 5.33) and HOG 2% (5.17), while OG 1% showed the most dramatic color change (∆*E* = 22.75), reinforcing the idea of high pigment mobility.

Cookie color also depends on the fat used (Table 3). Cookies made with butter remained light (L* = 58.62), while those made with OG 2% and hemp oil were significantly darker (L* = 45.15–47.24). HOG 2% of cookies maintained the highest yellow hue (b* = 23.2–20.2), while OG variants showed lower b* values.

Over 10 days, all cookies darkened slightly (increased L*) or shifted in chroma/hue values. The ∆*E*10 values (Figure 1, orange bars) show that the color change was highest in HOG 2% (4.48) and HOG 1% (5.02), suggesting that hydrogel matrices are more susceptible to oxidation and moisture-induced pigment shifts [17,18]. Lower ∆*E*10 was observed in OG 2% (2.64), followed by OG 1% (2.45), indicating improved color retention, likely due to the oil-phase encapsulation of pigments.

When comparing the cookie color to the butter-based reference (Figure 1, blue bars), the largest visual differences were seen in OG 1% (∆*E* = 13.03) and OG 2% (14.98), both exceeding the 5-unit threshold of visual perceptibility.

Despite offering good pigment retention over time, these variants differ notably in appearance, which could impact consumer acceptance [19]. Oleogel-based cookies, particularly OG 2%, exhibited improved color stability over storage and moderate fat-to-dough pigment integration. Hydroleogels, while promoting a vibrant initial color, showed higher instability during storage. Butter offered the most neutral and stable reference. These findings highlight a trade-off between pigment migration and color durability depending on fat structure.

### 2.3. Crispiness and Water Activity of Cookies

Crispiness and water activity (aw) are essential attributes determining the sensory appeal and storage stability of shortbread cookies. This section presents the results of instrumental crispiness analysis and water activity measurements of cookies prepared with various fat types, measured on the day of baking and after 10 days of storage (Table 4).

The type of fat used significantly affected the crispiness of cookies (*p* < 0.05), with observed differences directly linked to structural mechanisms. Butter-based cookies exhibited moderate initial crispiness (16.52 ± 1.14 N) that declined by 28% after 10 days (11.97 ± 0.40 N, Δ = 4.55 N), consistent with moisture migration in traditional high-fat systems. In contrast, cookies made with pure hemp oil showed inherently low crispiness (7.67 ± 1.23 N) due to the absence of a solid fat network, though they demonstrated relative stability during storage (Δ = 0.63 N).

Hydroleogel-based cookies achieved remarkable crispiness (HOG 2%: 45.67 ± 3.86 N)—nearly triple that of butter. This exceptional brittleness originates from the aqueous phase, which promotes extensive starch gelatinization and gluten network development during baking. The introduced water facilitates the hydration of wheat proteins and starch granules, creating a rigid, glassy matrix upon cooling that fractures cleanly under stress. However, this water-rich structure carries significant stability trade-offs: HOG 2% lost 25% of its crispiness over storage (Δ = 11.27 N) as moisture migrated through the crumb, while HOG 1% maintained its high value (36.10 ± 2.21 N), possibly due to structural reordering. Critically, the elevated water activity (aw = 0.39–0.61) in these systems approaches the mold growth threshold (aw > 0.60) [20], accelerating textural degradation and raising microbial safety concerns.

Oleogel-based cookies displayed the lowest crispiness values (OG 2%: 2.27 ± 0.28 N), attributed to their unique microstructure. The freeze-dried and re-homogenized agarose forms particulate networks rather than continuous matrices, creating fracture points that dissipate energy. These systems essentially “lubricate” the starch–gluten interface rather than reinforcing it, yielding a tender, crumbly texture distinct from brittle crispness. Despite this sensory limitation, OGs demonstrated exceptional stability (OG 2%: Δ = 0.45 N), maintaining textural integrity better than both butter and HOGs. This is facilitated by their hydrophobic nature and lower water activity (aw = 0.12–0.16), which effectively retard moisture migration and staling reactions [21]. The inverse relationship between agarose concentration and aw in OGs (2% < 1%) confirms enhanced water immobilization in stronger gel networks [21,22].

Butter occupies an intermediate position, offering moderate crispiness but suffering significant storage losses. The results highlight a fundamental trade-off: hydroleogels maximize initial sensory appeal through water-mediated crispness but compromise shelf-life, while oleogels prioritize stability through moisture control at the expense of a brittle texture. This dichotomy underscores the importance of application-specific fat structuring, where OG 2% emerges as the optimal solution for products requiring an extended shelf-life and consistent texture, despite its lower initial crispiness. Future formulations might balance these properties through hybrid systems or complementary hydrocolloids that enhance crispness in low-moisture matrices [23].

### 2.4. Morphological Characteristics of Cookies

The morphological characteristics of cookies, including specific volume, spreadability, and total baking loss, are crucial indicators of structural integrity, dough behavior, and moisture retention. Table 5 presents the results obtained for cookies made with various fat types.

The highest specific volume was observed in cookies prepared with hemp oil (2.22 ± 0.07 cm^3^/g), followed by butter (1.51 ± 0.09 cm^3^/g). The lowest volumes were recorded in cookies made with structured fats, especially HOG 1% (1.13 ± 0.10 cm^3^/g) and OG 1% (1.12 ± 0.05 cm^3^/g). This reduction can be attributed to a denser dough structure and reduced aeration capacity of structured fats, as noted in recent work by Banas et al. [17] and Kumar et al. [16]. Structuring agents like agarose increase viscosity and limit air entrapment, affecting oven rise and crumb openness.

The greatest baking losses were recorded for HOG 2% (15.71 ± 0.67%) and HOG 1% (14.82 ± 0.74%), indicating substantial moisture release likely from the aqueous phase of the hydroleogels. Cookies with oleogels showed the lowest mass loss: OG 1% (4.07 ± 2.04%) and OG 2% (5.87 ± 0.20%), suggesting that oleogels were more efficient at retaining fat and water during baking. These findings are consistent with recent studies showing the stabilizing effect of oleogel networks on moisture retention [4,17].

As illustrated in Table 5, cookies made with butter and hemp oil were lighter and more expanded, while hydroleogel- and oleogel-based cookies were denser and narrower. This may be due to differences in moisture loss, which is closely linked to fat composition and matrix structure.

These findings align with recent research evaluating the morphological performance of bakery products containing oleogel-based fat replacers. Pang et al. [4] demonstrated that cookies formulated with rice-bran-oil-based oleogels structured with soy wax exhibited a significantly higher specific volume and lower baking loss compared to butter-based controls, while maintaining acceptable spreadability. These improvements were attributed to enhanced gas retention and an improved internal structure provided by the oleogel network.

Furthermore, a study by Banaś et al. [17] investigated the use of agar–rapeseed oil hydroleogels as solid fat substitutes in shortbread cookies. The research found that cookies made with these hydroleogels had a comparable spreadability and specific volume to those made with traditional fats, suggesting that hydrophilic gelators can effectively mimic the functional properties of conventional fats in cookie formulations.

These studies reinforce the potential of structured lipids, such as OG 2%, to successfully replicate the functional roles of traditional fats in dough expansion and moisture management during baking.

### 2.5. Volatiles Release from Different Fat Matrices

**Figure 2:**
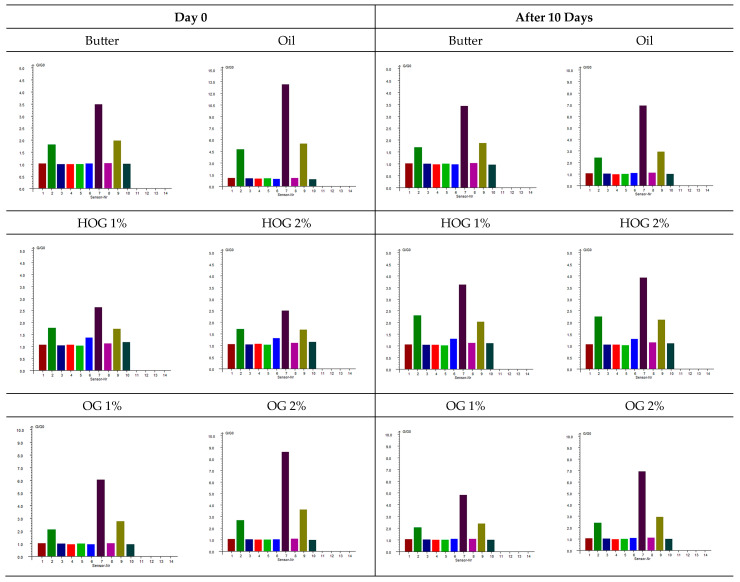
PEN3 sensor response diagram of shortbread cookies with different fat types. W1C (1): aromatics, benzene, toluene; W5S (2): nitrogen oxides, reactive gases; W3C (3): ammonia, amines; W6S (4): hydrogen, H_2_S; W5C (5): propane, methane, aliphatic hydrocarbons; W1S (6): methane (CH_4_); W1W (7): H_2_S, sulfur-containing VOCs; W2S (8): ethanol, CO; W2W (9): aromatics, organic sulfides; W3S (10): long-chain alkanes (e.g., hexane, pentane).

The exploitation of an electronic nose provides an objective assessment of the volatile compounds responsible for the aroma profile of cookies formulated with different fat types. Figure 2 presents the sensor responses for cookies at two time points: immediately after baking (day 0) and after 10 days of storage.

Butter-based cookies exhibited stable sensor responses over the storage period, indicating a consistent aroma profile. Notably, W7 (sulfur-organic compounds) showed high responses (3.42 ± 0.08 on day 0 and 3.41 ± 0.01 on day 10), suggesting the presence of sulfur-containing volatiles contributing to the characteristic buttery aroma. Hemp-oil-based cookies demonstrated significant changes in sensor responses over time. W2 (broad-range) showed a high initial response (4.79 ± 1.27 on day 0), which decreased substantially after 10 days (2.16 ± 0.19), indicating a loss of certain volatile compounds during storage. Additionally, W7 responses decreased from 13.57 ± 2.58 to 6.93 ± 1.14, suggesting a reduction in sulfur-containing volatiles.

Hydroleogel-based cookies (HOG 1% and HOG 2%) displayed relatively stable sensor responses over the storage period. For instance, HOG 1% showed W7 responses of 2.55 ± 0.10 on day 0 and 3.56 ± 0.11 on day 10, indicating a slight increase in sulfur-organic compounds. Sensor 6 (broad-methane) responses remained consistent, suggesting stability in aliphatic hydrocarbon content [24]. Oleogel-based cookies (OG 1% and OG 2%) exhibited moderate changes in sensor responses. OG 2% showed a decrease in W2 response from 3.47 ± 1.11 to 2.58 ± 0.48 over 10 days, indicating a reduction in certain volatile compounds. W7 responses also decreased from 10.10 ± 2.15 to 6.61 ± 1.78, suggesting a decline in sulfur-containing volatiles.

The analysis of variance (Table 6) revealed that the type of fat used in cookie formulations influenced the sensor responses across all sensors (*p* < 0.05). Storage time had a significant effect on sensors 2, 7, and 9, indicating changes in specific volatile compounds over time. The interaction between fat type and storage time was also notable for sensors 1–3 and 7–10, suggesting that the impact of storage on aroma profile varies depending on the fat type used.

The PEN3 sensor array data reveals distinct differences in volatile organic compound (VOC) formation among fat replacement systems, reflecting crucial alterations in thermally induced biochemical pathways and volatile releases dependent on structure [25,26]. Butter-based cookies exhibited characteristically high responses in W1C (aromatics) and W7 (sulfur compounds), reflecting dairy-specific Maillard reactions between milk proteins and reducing sugars that generate benzaldehyde derivatives and sulfur-containing volatiles like methanethiol [27,28,29,30].

This molecular pathway involves lysine/arginine residues forming Amadori rearrangement products that undergo cyclization and dehydration [28,29,30]. In contrast, W3C (ammonia/amines) responses confirmed the absence of protein-derived volatile precursors in plant-based systems, as the thermal deamination of asparagine/glutamine residues cannot occur without dairy proteins [31,32].

Hemp oil systems showed fundamentally different profiles, with pure oil (O) displaying intense initial responses in W5S (nitrogen oxides/reactive gases) and W7, indicating the rapid lipid oxidation of PUFAs generating hexanal and reactive aldehydes [31]. The 49% decrease in W7 response (13.57→6.93) over storage signals the progressive degradation of desirable nutty notes into stale aromas.

Structured systems modified this degradation: OG 2% demonstrated superior oxidative stability with smaller W7 reductions (10.10→6.61), preserving terpenes captured by W2W (sulfides), while HOGs maintained stable but distinct profiles with elevated W5C (aliphatic hydrocarbons) responses, indicating the hydrogel entrapment of lipid-derived volatiles.

Agarose’s molecular entrapment efficacy was evidenced by systematically reduced volatile release with increasing concentration. Hydrogen bonding between agarose chains creates a 3D network restricting diffusion [33], with denser 2% matrices showing enhanced retention. Critically, W1C responses confirmed that all plant-based systems lacked dairy-type Maillard aromatics, regardless of matrix modification—an irreplaceable sensory limitation. The convergence of hydroleogel and oleogel profiles at equivalent concentrations (e.g., HOG 1% vs. OG 1% W5C: 1.34 vs. 0.95) indicates that preserved polymer architecture post-lyophilization governs volatile mobility more than hydration state.

Butter’s stable fingerprint correlates with consistent “rich, creamy” notes [34], while pure hemp oil’s volatile decay manifests as a progression from initial “herbal” to “rancid”. HOGs’ stability suggests muted aroma development due to aqueous-phase entrapment, whereas OG 2% best preserves the intended “nutty” hemp character during shelf life. This is corroborated by OG 2%’s minimal changes in W3S (alkanes), indicating the effective encapsulation of oxidation-sensitive PUFAs.

The e-nose conclusively identifies OG 2% as maintaining the most stable hemp-like volatile fingerprint during storage, with 34% less aroma decay than pure hemp oil. HOGs preserve a different (water-moderated) profile, while butter remains sensorially distinct due to irreplaceable dairy protein volatiles. This stability differential reflects inherent antioxidant properties in dairy systems versus the oxidative susceptibility of plant-based PUFAs [35], with agarose structuring significantly mitigating degradation in OG 2%.

The optimal volatile release from 1% agarose systems reflects balanced polymer network porosity, while denser 2% matrices may over-restrict diffusion. These findings establish electronic nose technology as a powerful tool for quantifying butter replacement effectiveness, achieving >90% classification accuracy as demonstrated by Rivai and Aulia [36], with hydroleogels showing particular promise for aroma preservation [21,37,38].

## 3. Materials and Methods

### 3.1. Raw Materials

The raw materials used for the preparation of shortbread cookies included butter with 82% fat content (Mleczna Dolina, Sulechów, Poland) and cold-pressed hemp seed oil (BioNaturalis, Lublin, Poland). The choice of cold-pressed hemp seed oil was guided by its favorable functional and nutritional properties. Hemp seed oil is rich in polyunsaturated fatty acids, particularly essential omega-6 and omega-3 fatty acids in a beneficial 3:1–5:1 ratio [39]. This fatty acid profile supports improved texture and moisture retention in baked goods when compared to traditional fats. Moreover, studies have shown that emulsions or oleogels based on hemp seed oil can effectively substitute for conventional fats in cookies while maintaining desirable rheological and sensory qualities [13]. Nutritionally, hemp oil contains phytosterols and tocopherols, which are associated with cholesterol-lowering and antioxidant effects, thus enhancing the health profile of the final product. Together, these characteristics made cold-pressed hemp seed oil an ideal choice for formulating shortbread cookies with improved nutritional value, appealing texture, and functional stability [40]. As dry ingredients, type 480 wheat flour (Szymanowska, Polskie Młyny, Poland) and icing sugar were applied. Icing sugar was prepared by mechanically grinding refined white sugar (Cukier Polski S.A.,Toruń, Poland).

Agarose (PRONA basic LE, PRONA GmbH, Legden, Germany) was used as the sole structuring agent for both hydroleogels and oleogels. Agarose is a natural, linear polysaccharide extracted from red seaweeds such as *Gelidium* and *Gracilaria*. It is composed of alternating units of β-D-galactose and 3,6-anhydro-α-L-galactose (agarobiose), which together form the repeating disaccharide units of its backbone. Agarose is the primary gelling component of agar and is responsible for its unique gel-forming properties at low concentrations. The gelation of agarose occurs through the formation of double helices stabilized by hydrogen bonds, which then aggregate into a three-dimensional network upon cooling. This thermoreversible gelation mechanism produces transparent and firm gels with tunable rheological properties depending on the concentration, temperature, and molecular weight [41]. Due to its non-toxic and biocompatible nature, agarose is widely used in biomedical and food applications. In food systems, it serves as a clean-label structure-forming agent, enabling the development of hydrogel or oleogel systems with desirable texture and stability. Moreover, compared to agar, agarose contains fewer charged groups (such as sulphates), which contributes to stronger gelation and lower syneresis under comparable conditions [42].

All materials were purchased from local Polish retail distributors. Butter was used as the reference fat in the control cookie formulation, while the hemp oil and its structured forms were tested as alternative fat sources.

### 3.2. Preparation of Hydroleogels and Oleogels

Agarose hydrogels were prepared at two concentrations: 1% and 2% (*w/w*). Appropriate amounts of agarose (PRONA basic LE) were weighed into laboratory flasks and dissolved in distilled water. These concentrations were selected based on both practical considerations and manufacturer recommendations, which suggest using agarose up to 2% *w/w*. The choice was also inspired by previous work by K. Banaś [17], who used 1% and 3% concentrations of agar. However, agarose forms significantly stiffer gels at the same concentrations, and preliminary tests showed that 3% agarose resulted in a very rigid structure unsuitable for food applications. Therefore, 1% and 2% concentrations were selected as optimal to ensure oil entrapment while maintaining a stable yet soft and food-compatible texture. The mixtures were sterilized using an autoclave (Microjet autoclave, BiotoolSwiss GmbH, Switzerland) to ensure full solubilization and microbial safety. After autoclaving, the 1% agarose solution was cooled to 45 °C and the 2% solution to 55 °C, respectively. In parallel, cold-pressed hemp seed oil was heated to the same target temperatures: 45 °C for the 1% system and 55 °C for the 2% system. Once both phases reached the desired temperature, the aqueous agarose solution was added to the oil phase in a 1:1 (*w/w*) ratio. The mixture was homogenized for 1 min at 10,000 rpm using a high-speed mechanical homogenizer (Yellow line DI 18 Basic, IKA^®^-Werke GmbH & Co. KG, Königswinter, Germany) to obtain a uniform emulsion. The resulting hydroleogels were left to cool and solidify at room temperature.

To obtain oleogels, the hydroleogels were subjected to a freeze-drying process. Samples were frozen at −70 °C and subsequently lyophilized for 23 h (lyophilizer Alpha 1-4 LSCplus, Martin Christ Gefriertrocknungsanlagen GmbH, Osterode am Harz, Germany). The freeze-dried materials were mechanically ground and redispersed by homogenization for 1 min at 10,000 rpm using the same homogenizer, yielding structured oleogels.

### 3.3. Preparation of Shortbread Cookies

Shortbread dough was prepared using a standardized formulation based on a flour/fat/sugar weight ratio of 3:2:1. Each batch contained 200 g of the fat component, which varied depending on the experimental group: butter (control), cold-pressed hemp seed oil, hydroleogels (1% and 2% agarose), or oleogels (1% and 2% agarose). The remaining ingredients, wheat flour and icing sugar, were kept constant across all formulations.

The ingredients were combined and kneaded manually until a uniform dough was formed. The dough was then refrigerated at 4 °C for 1 h to stabilize the structure before further processing. After cooling, the dough was rolled out to a uniform thickness of 8 mm and cut into cookie shapes using a standard cookie cutter. The cookies were baked in a convection oven (HENDI, NANO Nanotechnology ovens, Robakowo, Poland) at 180 °C for 20 min. After baking, cookies were left to cool at room temperature prior to further analysis.

### 3.4. Physicochemical Parameters of Doughs and Cookies

#### 3.4.1. Texture Analysis of Raw Shortbread Dough

The texture of raw shortbread doughs was analyzed using a double compression test performed on a texture analyzer (FC200STAV200, AXIS, Gdansk, Poland), equipped with a cylindrical probe of 50 mm diameter. Dough samples were shaped into cylinders (20 mm height × 20 mm diameter) and conditioned at room temperature. Measurements were conducted at two time points: 5 min and 15 min after removal from refrigeration (4 °C). The compression was applied to 40% strain, with a test speed of 100 mm/min and a rest time of 3 s between cycles. For each fat type, four independent replicates were analyzed. The following parameters were recorded: hardness, springiness, cohesiveness, gumminess, and resilience.

#### 3.4.2. Crispiness Analysis of Shortbread Cookies

The crispiness of baked cookies was evaluated using a three-point bending test on a texture analyzer (FC020STAV500, AXIS, Poland) with a point load attachment. The probe descended at a speed of 150 mm/s to a depth of 20 mm. Crispiness was determined based on the maximum force at fracture (N). Measurements were performed at two storage time points: 1 h after baking and after 10 days of storage. Each sample was tested in four replicates per fat variant.

#### 3.4.3. Color Analysis

Color measurements were performed using a Minolta CR-310 colorimeter (Konica Minolta, Tokyo, Japan) calibrated against a standard white plate. The CIELab color parameters were recorded: L* (lightness), a* (red–green), and b* (yellow–blue). From these, hue angle (h°) and chroma (C*) were also calculated. Color analysis was conducted for fat samples, raw cookie doughs, cookies on the day of baking, and cookies after 10 days of storage. Each sample was measured in triplicate. Measurements were taken under standard laboratory lighting conditions, and the results were averaged for statistical comparison.

To assess color changes, Δ*E* (total color difference) was calculated between dough and the corresponding fat sample to evaluate pigment migration and dispersion. Additionally, Δ*E* values were determined between each cookie variant and the reference cookie made with butter (B) to evaluate the visual impact of fat substitution. Further Δ*E* values were used to compare the change in cookie color over storage for each fat variant.

Δ*E* was calculated using the standard CIELab Formula (1):

(1) $$∆E=\sqrt{{\left(∆L^{*}\right)}^{2}+{\left(∆a^{*}\right)}^{2}+{\left(∆b^{*}\right)}^{2}}$$

#### 3.4.4. Morphological Characterization

The visual appearance of cookies was documented using a Samsung Galaxy S23, (Samsung, Suwon, South Korea) smartphone equipped with a triple-lens camera system: 50 MP wide-angle (f/1.8, 24 mm), 10 MP telephoto (f/2.4, 70 mm, 3× optical zoom), and 12 MP ultra-wide (f/2.2, 13 mm). Photographs were taken under natural daylight with a fixed focal length and 4:3 aspect ratio.

The specific volume of the cookies [cm^3^/g] was determined using the millet seed displacement method, as described by Banaś et al. [17]. Measurements were performed in triplicate for each sample group, using varying numbers of cookies (3 to 5 per repetition) to ensure consistency and reduce measurement error. The specific volume is calculated using Formula (2):

(2) $$\nu =\frac{V}{w}$$

where

*ν*—specific volume [cm^3^/g];

*V*—measured volume of the cookies [cm^3^];

*w*—weight of the cookies [g].

The spreadability of the cookies was assessed by measuring their diameter before and after baking. For each fat type, five cookies were selected, and five perpendicular diameter measurements were taken for each cookie using a digital caliper with 0.01 mm precision. Spreadability was expressed as the percentage change in diameter after baking relative to the pre-baking dimension.

Total baking loss [%] was calculated based on the mass difference of cookies before and after baking, following Formula (3):

(3) $$Baking\;loss=\frac{m_{before}-m_{after}}{m_{before}}*100\%,$$

where *m_befor__e_* and *m_after_* represent the weight of the dough before and after baking, respectively. Each fat variant was tested in 10 repetitions.

#### 3.4.5. Electronic Nose Analysis

The volatile profile of shortbread cookies was analyzed using an electronic nose system PEN 3 (AIRSENSE Analytics GmbH, Schwerin, Germany), equipped with an array of metal oxide semiconductor (MOS) sensors sensitive to a wide range of volatile organic compounds. Data acquisition and analysis were performed using the WinMuster PEN 1.6.2.25 software (AIRSENSE Analytics, Schwerin, Germany). The PEN3 sensor array provided comprehensive volatile profiling across ten distinct chemical detection zones, encompassing aromatic compounds (W1C, W2W), nitrogen-containing volatiles (W5S, W3C), sulfur compounds (W6S, W1W), hydrocarbons of varying chain lengths (W5C, W1S, W3S), and alcohol–carbonyl compounds (W2S). This sensor configuration enabled the detection of volatile compounds spanning the complete range of thermally induced reactions occurring during shortbread baking, including Maillard reaction products, lipid oxidation derivatives, and protein thermal degradation compounds.

Each sample was measured in triplicate at two time points: 1 h after baking and after 10 days of storage. Prior to measurement, samples were placed in 100 mL sealed glass vials and equilibrated at room temperature. The system was operated under ambient laboratory conditions according to the manufacturer’s recommendations. The generated sensor responses were used for comparative evaluation of the aromatic fingerprint between fat types and over storage time.

#### 3.4.6. Water Activity Measurement

The water activity of the cookies was measured on the day of baking and after 10 days of storage using a water activity analyzer, AquaLab (Series 3TE, Decagon Devices, Inc., USA). Each sample was analyzed in triplicate. The measurements were conducted at ambient laboratory temperature (approximately 23 ± 1 °C). Values were recorded once the device reached equilibrium for each sample.

#### 3.4.7. Statistical Analysis

All statistical analyses were performed using Statistica 13.3 software (StatSoft Inc., Tulsa, OK, USA). The results were expressed as means ± standard deviation. Depending on the dataset structure, one-way or multifactor analysis of variance (ANOVA) was applied to assess the significance of differences between experimental groups. When statistically significant effects were found (*p* ≤ 0.05), Tukey’s honest significant difference (HSD) post hoc test was used for pairwise comparisons.

## 4. Conclusions

This study confirms that structuring cold-pressed hemp oil using agarose as the sole gelator is not only feasible but also effective for producing high-quality shortbread cookies. Hydroleogels and oleogels based on agarose proved to be capable fat replacers, delivering improved nutritional profiles while maintaining key technological and sensory properties. Among the tested variants, OG 2% showed the best overall performance in terms of texture, morphology, aroma stability, and water activity. PEN3 electronic nose analysis provides comprehensive insights into the volatile behavior of alternative fat systems in shortbread cookie applications. The fundamental differences between dairy and plant-based systems, particularly regarding protein-derived volatiles, represent inherent challenges that cannot be fully addressed through matrix engineering alone. However, gel-based systems demonstrate significant potential for enhancing volatile complexity compared to pure hemp oil while providing processing advantages and improved functionality. The convergence of hydroleogel and oleogel volatile profiles at equivalent agarose concentrations simplifies formulation decisions, while the systematic concentration effects provide predictable optimization pathways. These findings demonstrate that successful fat replacement requires understanding molecular-level interactions between lipid matrices, protein components, and polysaccharide networks. The inability to replicate dairy-specific Maillard reaction products through matrix modification highlights the molecular specificity of protein-derived volatile formation pathways. Future developments in fat replacement technology must address these molecular limitations through targeted protein incorporation or enzymatic flavor enhancement strategies. The use of agarose as a natural, safe, and functional gelator presents an attractive strategy for formulating plant-based bakery products with reduced saturated fat content. Future research should explore the further optimization of agarose concentration and evaluate its potential in other food applications.

## Figures and Tables

**Table 1:** Textural parameters of raw shortbread doughs formulated with different fat types were measured after 5 and 15 min of conditioning at room temperature.

Fat type	T [min]	Hardness [N]	Cohesiveness	Springiness	Gumminess [N]	Resilience
B	5	37.30 ± 1.92 ^e,B^	0.09 ± 0.01 ^a,A^	0.37 ± 0.15 ^c,B^	3.47 ± 0.55 ^d,B^	1.86 ± 0.16 ^bc,B^
	15	16.33 ± 0.81 ^e,A^	0.14 ± 0.00 ^a,B^	0.09 ± 0.01 ^c,A^	2.21 ± 0.08 ^d,A^	0.17 ± 0.01 ^bc,A^
O	5	3.87 ± 0.95 ^a,B^	0.06 ± 0.01 ^a,A^	0.26 ± 0.04 ^b,B^	0.21 ± 0.03 ^a,B^	0.65 ± 0.29 ^ab,B^
	15	3.12 ± 0.81 ^a,A^	0.04 ± 0.01 ^a,B^	0.16 ± 0.02 ^b,A^	0.14 ± 0.05 ^a,A^	0.39 ± 0.04 ^ab,A^
HOG 1%	5	5.65 ± 0.51 ^ab,B^	0.27 ± 0.00 ^b,A^	0.35 ± 0.03 ^d,B^	1.54 ± 0.12 ^c,B^	0.29 ± 0.02 ^a,B^
	15	3.25 ± 0.78 ^ab,A^	0.34 ± 0.12 ^b,B^	0.37 ± 0.02 ^d,A^	1.05 ± 0.08 ^c,A^	0.29 ± 0.06 ^a,A^
HOG 2%	5	8.35 ± 1.61 ^c,B^	0.23 ± 0.02 ^b,A^	0.32 ± 0.04 ^d,B^	1.86 ± 0.27 ^c,B^	0.27 ± 0.04 ^a,B^
	15	4.22 ± 0.03 ^c,A^	0.27 ± 0.01 ^b,B^	0.34 ± 0.00 ^d,A^	1.13 ± 0.07 ^c,A^	0.27 ± 0.03 ^a,A^
OG 1%	5	6.22 ± 0.36 ^bc,B^	0.09 ± 0.01 ^a,A^	0.09 ± 0.01 ^a,B^	0.55 ± 0.05 ^b,B^	0.30 ± 0.03 ^a,B^
	15	5.92 ± 0.12 ^bc,A^	0.10 ± 0.00 ^a,B^	0.10 ± 0.01 ^a,A^	0.57 ± 0.01 ^b,A^	0.28 ± 0.10 ^a,A^
OG 2%	5	9.08 ± 0.48 ^d,B^	0.07 ± 0.00 ^a,A^	0.17 ± 0.01 ^ab,B^	0.65 ± 0.05 ^b,B^	1.07 ± 0.82 ^d,B^
	15	7.72 ± 0.44 ^d,A^	0.08 ± 0.00 ^a,B^	0.16 ± 0.01 ^ab,A^	0.58 ± 0.05 ^b,A^	1.67 ± 0.39 ^d,A^
Fat type		***	***	***	***	***
Time		***	*	***	***	*
Fat type × Time		***	ns	***	***	***

T [min]—conditioning time before texture analysis, B—butter (control sample), O—cold-pressed hemp oil, HOG 1%—hydroleogel containing 1% agarose and cold-pressed hemp oil, HOG 2%—hydroleogel containing 2% agarose and cold-pressed hemp oil, OG 1%—oleogel containing 1% agarose and cold-pressed hemp oil, OG 2%—oleogel containing 2% agarose and cold-pressed hemp oil. Lowercase letters indicate statistically significant differences between fat types (*p* ≤ 0.05); uppercase letters indicate statistically significant differences between conditioning times (*p* ≤ 0.05). ***—statistically different at *p* < 0.001; *—statistically different for *p* < 0.05; ns—not significant.

**Table 2:** Color parameters (L*, a*, b*, C, h) of different fats and raw cookie doughs prepared with these fats.

**Different Types of Fats**						
	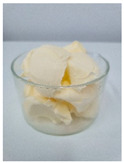	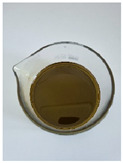	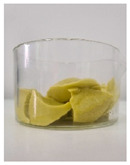	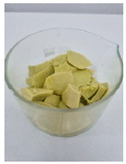	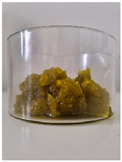	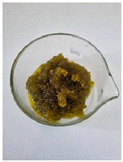
**Fat type**	B	O	HOG 1%	HOG 2%	OG 1%	OG 2%
L*	92.58 ± 0.17 ^c^	44.79 ± 0.97 ^a^	54.75 ± 3.53 ^b^	55.68 ± 1.58 ^b^	56.81 ± 1.70 ^b^	53.89 ± 0.20 ^b^
a*	−4.04 ± 0.02 ^b^	−5.29 ± 0.39 ^ab^	−1.30 ± 0.14 ^c^	−1.38 ± 0.10 ^c^	−5.99 ± 1.22 ^a^	−4.74 ± 0.79 ^ab^
b*	21.75 ± 0.42 ^a^	21.26 ± 0.67 ^a^	26.85 ± 1.74 ^ab^	27.43 ± 1.24 ^ab^	45.70 ± 6.61 ^c^	37.43 ± 9.97 ^bc^
C	24.57 ± 0.42 ^a^	38.17 ± 0.92 ^cd^	41.31 ± 4.87 ^d^	39.42 ± 0.86 ^d^	34.03 ± 1.23 ^bc^	32.40 ± 0.86 ^b^
h	99.13 ± 0.17 ^b^	99.83 ± 0.74 ^b^	100.25 ± 0.62 ^b^	100.15 ± 1.27 ^b^	96.50 ± 0.54 ^a^	96.65 ± 1.15 ^a^
**Raw dough prepared with different fat types**						
	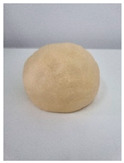	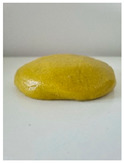	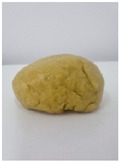	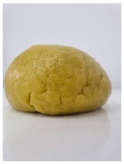	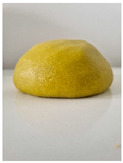	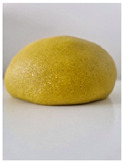
**Fat type**	B	O	HOG 1%	HOG 2%	OG 1%	OG 2%
L*	80.45 ± 2.04 ^d^	47.24 ± 0.77 ^a^	57.23 ± 1.41 ^c^	53.69 ± 0.23 ^b^	53.57 ± 0.84 ^b^	55.76 ± 0.89 ^bc^
a*	−0.67 ± 0.15 ^d^	2.53 ± 0.45 ^e^	−3.53 ± 0.11 ^b^	−3.05 ± 0.07 ^c^	−4.20 ± 0.08 ^a^	−3.88 ± 0.09 ^ab^
b*	22.81 ± 0.28 ^b^	17.13 ± 0.35 ^a^	24.87 ± 0.95 ^c^	23.10 ± 0.37 ^b^	23.29 ± 0.46 ^b^	24.49 ± 0.39 ^c^
C	27.47 ± 0.16 ^a^	26.35 ± 0.39 ^a^	38.24 ± 1.40 ^c^	36.10 ± 0.86 ^b^	36.75 ± 0.63 ^bc^	38.22 ± 0.48 ^c^
h	91.40 ± 0.29 ^b^	83.38 ± 1.33 ^a^	96.08 ± 0.22 ^c^	95.70 ± 0.22 ^c^	97.75 ± 0.13 ^d^	96.75 ± 0.10 ^cd^

Lowercase letters indicate statistically significant differences among fat types (*p* ≤ 0.05).

**Table 3:** Color parameters (L*, a*, b*, C*, h˚) of cookies prepared with different fat types and measured at 0 and 10 days of storage.

Fat Type	Time	L*	a*	b*	C	h
**B**	0	58.62 ± 0.76 ^c,A^	6.19 ± 0.26 ^d^	21.21 ± 0.16 ^b^	30.92 ± 0.19 ^d,B^	77.18 ± 0.57 ^a,A^
	10	61.08 ± 1.03 ^c,B^	5.27 ± 0.34 ^d^	21.17 ± 0.24 ^b^	29.87 ± 0.41 ^d,A^	78.83 ± 0.74 ^a,B^
**O**	0	47.24 ± 0.77 ^b,A^	2.53 ± 0.45 ^b^	17.13 ± 0.35 ^a^	26.35 ± 0.39 ^a,B^	83.38 ± 1.33 ^c,A^
	10	48.96 ± 0.89 ^b,B^	2.62 ± 0.26 ^b^	18.40 ± 0.28 ^a^	28.27 ± 0.89 ^a,A^	83.70 ± 0.45 ^c,B^
**HOG 1%**	0	60.57 ± 1.85 ^d,A^	−1.09 ± 0.59 ^a^	23.52 ± 0.30 ^b^	33.86 ± 0.95 ^cd,B^	92.00 ± 1.15 ^e,A^
	10	62.25 ± 1.19 ^d,B^	−0.44 ± 0.31 ^a^	19.50 ± 0.08 ^b^	26.10 ± 0.31 ^cd,A^	91.03 ± 0.74 ^e,B^
**HOG 2%**	0	60.81 ± 0.82 ^d,A^	−0.96 ± 0.37 ^a^	23.22 ± 0.23 ^b^	33.16 ± 0.61 ^d,B^	91.80 ± 0.74 ^e,A^
	10	63.57 ± 1.43 ^d,B^	−0.49 ± 0.42 ^a^	20.20 ± 1.48 ^b^	27.00 ± 2.13 ^d,A^	91.15 ± 1.10 ^e,B^
**OG 1%**	0	46.55 ± 1.22 ^b,A^	2.52 ± 0.65 ^b^	18.05 ± 0.67 ^a^	28.48 ± 0.84 ^bc,B^	83.85 ± 1.80 ^d,A^
	10	48.62 ± 0.43 ^b,B^	1.63 ± 0.21 ^b^	18.86 ± 0.22 ^a^	29.22 ± 0.28 ^bc,A^	86.20 ± 0.56 ^d,B^
**OG 2%**	0	45.15 ± 0.49 ^a,A^	3.61 ± 0.26 ^c^	15.86 ± 3.62 ^a^	28.41 ± 0.15 ^ab,B^	81.15 ± 0.65 ^b,A^
	10	46.17 ± 0.16 ^a,B^	3.32 ± 0.03 ^c^	17.88 ± 0.10 ^a^	28.36 ± 0.15 ^ab,A^	81.90 ± 0.08 ^b,B^
fat type		***	***	***	***	***
time		***	ns	ns	***	*
fat type × time		ns	***	***	***	***

Time—storage time in days (0 = day of baking; 10 = after 10 days of storage). Lowercase letters indicate statistically significant differences among fat types (*p* ≤ 0.05); uppercase letters indicate statistically significant differences for sample in time (*p* ≤ 0.05). ***—statistically different at *p* < 0.001; *—statistically different for *p* < 0.05; ns—not significant.

**Table 4:** Crispiness and water activity of shortbread cookies formulated with different fat types were measured on the day of baking and after 10 days of storage.

Fat Type	Time	Crispiness [N]	Change in Crispiness in Time [N]	Water Activity
**B**	0	16.52 ± 1.14 ^c,B^	4.55 ± 1.54 ^ab^	0.16 ± 0.01 ^b,A^
	10	11.97 ± 0.40 ^c,A^		0.18 ± 0.01 ^b,B^
**O**	0	7.67 ± 1.23 ^b,B^	0.63 ± 1.86 ^a^	0.06 ± 0.00 ^a,A^
	10	7.03 ± 1.55 ^b,A^		0.08 ± 0.01 ^a,AB^
**HOG 1%**	0	35.07 ± 3.84 ^d,B^	−1.03 ± 4.69 ^a^	0.61 ± 0.02 ^d,A^
	10	36.10 ± 2.21 ^d,A^		0.53 ± 0.01 ^d,B^
**HOG 2%**	0	45.67 ± 3.86 ^e,B^	11.27 ± 7.70 ^b^	0.39 ± 0.07 ^c,A^
	10	34.40 ± 4.33 ^e,A^		0.47 ± 0.01 ^c,B^
**OG 1%**	0	3.43 ± 0.77 ^ab,B^	−0.50 ± 1.15 ^a^	0.12 ± 0.01 ^b,A^
	10	3.93 ± 0.78 ^ab,A^		0.15 ± 0.01 ^b,B^
**OG 2%**	0	2.27 ± 0.28 ^a,B^	0.45 ± 0.61 ^a^	0.13 ± 0.01 ^b,A^
	10	1.82 ± 0.76 ^a,A^		0.16 ± 0.01 ^b,B^
**Fat type**		***	-	***
**Time**		***	-	*
**Fat type × Time**		***	-	***

Time—storage time in days (0 = day of baking; 10 = after 10 days of storage), Lowercase letters indicate statistically significant differences between fat types (*p* ≤ 0.05); uppercase letters indicate statistically significant differences between conditioning times for particular sensors (*p* ≤ 0.05). ***—statistically different at *p* < 0.001; *—statistically different for *p* < 0.05.

**Table 5:** Morphological characteristics of shortbread cookies prepared with different fat types.

Appearance	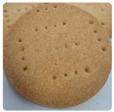	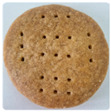	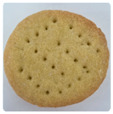	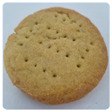	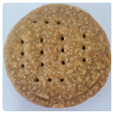	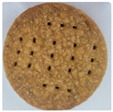
Fat Type	B	O	HOG 1%	HOG 2%	OG 1%	OG 2%
**Specific volume [cm^3^/g]**	1.51 ± 0.09 ^b^	2.22 ± 0.07 ^c^	1.13 ± 0.10 ^a^	1.31 ± 0.06 ^ab^	1.12 ± 0.05 ^a^	1.23 ± 0.09 ^a^
**Spreadability** **[%]**	2.83 ± 0.64 ^c^	2.83 ± 0.64 ^c^	−1.85 ± 0.68 ^a^	−1.59 ± 0.27 ^a^	−1.00 ± 0.57 ^ab^	−0.47 ± 0.29 ^b^
**Total baking loss** **[%]**	9.00 ± 0.34 ^c^	8.03 ± 1.07 ^c^	14.82 ± 0.74 ^d^	15.71 ± 0.67 ^d^	4.07 ± 2.04 ^a^	5.87 ± 0.20 ^b^

Lowercase letters indicate statistically significant differences between fat types (*p* ≤ 0.05).

**Table 6:** PEN 3 sensor values and statistical analysis in shortbread cookies 1 h after baking (day 0) and after 10 days of storage.

	Day 0						
Fat Type		Butter	Oil	HOG 1%	HOG 2%	OG 1%	OG 2%
**Sensors [G_0_/G]**	**(1)**	0.99 ± 0.01 ^c^	0.96 ± 0.00 ^b^	0.94 ± 0.01 ^a^	0.95 ± 0.01 ^a^	0.98 ± 0.00 ^b^	0.95 ± 0.01 ^a^
	**(2)**	1.76 ± 0.09 ^a,B^	4.79 ± 1.27 ^c,B^	1.74 ± 0.04 ^ab,B^	1.76 ± 0.06 ^ab,B^	2.13 ± 0.01 ^ab,B^	3.47 ± 1.11 ^bc,B^
	**(3)**	1.00 ± 0.01 ^c^	0.99 ± 0.00 ^c^	0.96 ± 0.00 ^a^	0.97 ± 0.00 ^a^	1.00 ± 0.00 ^bc^	0.98 ± 0.00 ^b^
	**(4)**	0.98 ± 0.03 ^ab^	0.95 ± 0.02 ^a^	1.07 ± 0.01 ^b^	1.14 ± 0.11 ^c^	0.96 ± 0.00 ^ab^	0.99 ± 0.01 ^ab^
	**(5)**	1.00 ± 0.00 ^bc^	1.00 ± 0.00 ^c^	0.98 ± 0.00 ^a^	0.98 ± 0.00 ^a^	1.00 ± 0.00 ^b^	1.00 ± 0.00 ^b^
	**(6)**	0.98 ± 0.06 ^ab^	0.95 ± 0.02 ^a^	1.34 ± 0.05 ^c^	1.25 ± 0.05 ^c^	0.95 ± 0.00 ^b^	1.04 ± 0.01 ^b^
	**(7)**	3.42 ± 0.08 ^a,B^	13.57 ± 2.58 ^c,B^	2.55 ± 0.10 ^a,B^	2.74 ± 0.09 ^a,B^	6.17 ± 0.22 ^ab,B^	10.10 ± 2.15 ^bc,B^
	**(8)**	1.02 ± 0.03 ^a^	1.09 ± 0.00 ^a^	1.12 ± 0.03 ^b^	1.11 ± 0.00 ^b^	1.03 ± 0.00 ^a^	1.11 ± 0.02 ^b^
	**(9)**	1.96 ± 0.04 ^a,B^	5.20 ± 0.63 ^b,B^	1.71 ± 0.04 ^a,B^	1.77 ± 0.04 ^a,B^	2.84 ± 0.10 ^a,B^	4.13 ± 0.74 ^b,B^
	**(10)**	0.99 ± 0.03 ^b^	0.93 ± 0.01 ^a^	1.17 ± 0.02 ^c^	1.13 ± 0.02 ^c^	0.95 ± 0.00 ^b^	0.98 ± 0.01 ^b^
	**After 10 days**						
**Fat type**		**B**	**O**	**HOG 1%**	**HOG 2%**	**OG 1%**	**OG 2%**
**Sensors[G_0_/G]**	**(1)**	0.98 ± 0.00 ^c^	0.99 ± 0.01 ^b^	0.94 ± 0.00 ^a^	0.95 ± 0.00 ^a^	0.96 ± 0.00 ^b^	0.95 ± 0.00 ^a^
	**(2)**	1.72 ± 0.04 ^a,A^	2.16 ± 0.19 ^c,A^	2.27 ± 0.07 ^ab,A^	1.97 ± 0.08 ^ab,A^	2.12 ± 0.21 ^ab,A^	2.58 ± 0.48 ^bc,A^
	**(3)**	1.00 ± 0.00 ^c^	1.00 ± 0.01 ^c^	0.97 ± 0.00 ^a^	0.97 ± 0.00 ^a^	0.98 ± 0.00 ^bc^	0.98 ± 0.00 ^b^
	**(4)**	0.98 ± 0.01 ^ab^	0.94 ± 0.02 ^a^	1.05 ± 0.00 ^b^	1.03 ± 0.01 ^c^	1.00 ± 0.01 ^ab^	0.99 ± 0.01 ^ab^
	**(5)**	1.00 ± 0.00 ^bc^	1.01 ± 0.00 ^c^	0.98 ± 0.00 ^a^	0.99 ± 0.00 ^a^	0.99 ± 0.00 ^b^	0.99 ± 0.00 ^b^
	**(6)**	1.01 ± 0.04 ^ab^	0.90 ± 0.05 ^a^	1.33 ± 0.03 ^c^	1.23 ± 0.06 ^c^	1.10 ± 0.05 ^b^	1.09 ± 0.02 ^b^
	**(7)**	3.41 ± 0.01 ^a,A^	6.93 ± 1.14 ^c,A^	3.56 ± 0.11 ^a,A^	3.32 ± 0.04 ^a,A^	4.99 ± 1.85 ^ab,A^	6.61 ± 1.78 ^bc,A^
	**(8)**	1.04 ± 0.02 ^a^	1.03 ± 0.02 ^a^	1.15 ± 0.00 ^b^	1.11 ± 0.01 ^b^	1.10 ± 0.01 ^a^	1.14 ± 0.01 ^b^
	**(9)**	1.86 ± 0.01 ^a,A^	2.93 ± 0.32 ^b,A^	2.00 ± 0.03 ^a,A^	1.91 ± 0.01 ^a,A^	2.41 ± 0.55 ^a,A^	2.88 ± 0.52 ^b,A^
	**(10)**	1.00 ± 0.04 ^b^	0.91 ± 0.03 ^a^	1.12 ± 0.01 ^c^	1.08 ± 0.02 ^c^	1.02 ± 0.04 ^b^	1.03 ± 0.00 ^b^
	**Statistical analysis**						
		**Fat type**		**Time**		**Fat type × Time**	
**Sensors**	**(1)**	***		ns		***	
	**(2)**	***		*		**	
	**(3)**	***		ns		***	
	**(4)**	***		ns		ns	
	**(5)**	***		ns		***	
	**(6)**	***		ns		ns	
	**(7)**	***		***		***	
	**(8)**	***		ns		***	
	**(9)**	***		***		***	
	**(10)**	***		ns		***	

(1): Aromatics, benzene, toluene; (2): nitrogen oxides, reactive gases; (3): ammonia, amines; (4): hydrogen, H_2_S; (5): propane, methane, aliphatic hydrocarbons; (6): methane (CH_4_); (7): H_2_S, sulfur-containing VOCs; (8): ethanol, CO; (9): aromatics, organic sulfides; (10): long-chain alkanes (e.g., hexane, pentane). Lowercase letters indicate statistically significant differences between fat types (*p* ≤ 0.05); uppercase letters indicate statistically significant differences in time (*p* ≤ 0.05). ***—statistically different at *p* < 0.001; **—statistically different at *p* < 0.01; *—statistically different for *p* < 0.05; ns—not significant.

## Data Availability

All data are included in the article.
